# Beyond the Biomarkers: Gestation-Dependent Roles of sFLT-1 and PlGF in Human and Non-Human Mammalian Placental Development and Function

**DOI:** 10.3390/cells15191817

**Published:** 2026-10-06

**Authors:** Zoe Byrne, Sonja Brennan, David Watson, Donna Rudd, Yoga Kandasamy

**Affiliations:** 1 Maternal Fetal Medicine Unit, Obstetrics & Gynaecology Department, Townsville Hospital and Health Service, 100 Angus Smith Drive, Douglas, Townsville, QLD 4810, Australia; 2 College of Medicine and Dentistry, James Cook University, Townsville, QLD 4810, Australia; 3 Neonatal Intensive Care Unit, Townsville Hospital and Health Service, Townsville, QLD 4810, Australia

**Keywords:** placenta, soluble fms-like tyrosine kinase-1, sFLT-1, placental growth factor, PlGF, VEGF, trophoblast, angiogenesis, pre-eclampsia, fetal growth restriction

## Abstract

**Highlights:**

**What are the main findings?**

- The placental actions of soluble FLT1 (sFLT-1) and placental growth factor (PlGF) depend on gestation, compartment and isoform.
- Human observations and experimental manipulations provide complementary, but distinct, evidence of angiogenic regulation.

**What are the implications of the main findings?**

- The maternal sFLT-1/PlGF ratio does not directly measure local receptor activity or identify the initiating placental lesion.
- The compartmentalised angiogenic rheostat is an integrative framework, not an experimentally validated model.

**Abstract:**

Placental growth factor (PlGF) and soluble fms-like tyrosine kinase-1 (sFLT-1) are established biomarkers of placental dysfunction, but their functions extend beyond the maternal circulating ratio. This structured narrative review examines the FLT1–PlGF–vascular endothelial growth factor system across implantation, trophoblast differentiation, uterine vascular transformation, foetoplacental angiogenesis, exchange capacity development and late-gestational maturation. Human evidence is integrated with selected non-human mammalian studies according to gestational stage, cellular source, anatomical compartment, molecular isoform and placental architecture. We use a compartmentalised angiogenic rheostat as a qualitative organising framework, not an experimentally validated model. It integrates established ligand–receptor biology with human observations and experimental perturbations: PlGF modifies FLT1 signalling and VEGF-A availability, whereas membrane and soluble FLT1 influence ligand distribution and vascular responses. Experimental evidence supports physiological ligand restraint and the pathogenic effects of excessive sFLT-1; human associations do not invariably establish the initiating placental lesion. Pre-eclampsia, foetal growth restriction and invasive placentation illustrate distinct contexts in which angiogenic regulation may become maladaptive. The maternal sFLT-1/PlGF ratio is therefore interpreted as a circulating indicator of placental vascular stress rather than a direct measurement of local signalling.

## 1. Introduction

The mammalian vascular endothelial growth factor (VEGF) family is composed of five growth factors: VEGF-A, VEGF-B, placental growth factor (PlGF), VEGF-C, and VEGF-D [1]. PlGF and soluble fms-like tyrosine kinase-1 (sFLT-1) are best known as reciprocal maternal biomarkers of pre-eclampsia, yet both are normal components of placental development. PlGF was first isolated from human placenta [2]. FLT1 was identified as a high-affinity VEGF receptor, and sFLT-1 as an endogenously encoded soluble receptor that binds VEGF-family ligands [3,4,5]. Clinical interest followed evidence that excess placental sFLT-1 contributes to maternal endothelial dysfunction, hypertension and proteinuria, and that increasing sFLT-1 with falling PlGF can precede pre-eclampsia [6,7]. The sFLT-1/PlGF ratio now supports short-term risk assessment in suspected disease [8].

Both biomarkers are present in uncomplicated pregnancy, vary markedly with gestational age, and function within local placental compartments that are not fully reflected in maternal blood levels [9,10,11]. sFLT-1 is expressed during early trophoblast differentiation, whereas PlGF participates in endometrial, trophoblast, endothelial and immune communication [12,13,14]. Their effects depend on ligand concentration, receptor context, molecular isoform, extracellular retention and anatomical location.

Previous reviews have already distinguished angiogenic-factor activity within the placenta from that in the maternal circulation [15]. Recent syntheses address angiogenic factors in physiological and pathological pregnancy [16] and sFLT-1/PlGF as diagnostic and therapeutic targets in pre-eclampsia [17]. Our recent BJOG systematic review focuses specifically on the maternal ratio for distinguishing pathological foetal growth restriction (FGR) from constitutional smallness [18]. These contributions establish important conceptual and clinical foundations.

The present structured narrative review complements this literature by organising the evidence from implantation to term and integrating developmental timing, compartmentalisation, isoform biology and cross-species experimental findings. We use the term compartmentalised angiogenic rheostat for this qualitative synthesis; it does not denote a newly demonstrated regulatory mechanism or a validated quantitative model. Its contribution is to connect established components, distinguish human observations from experimental perturbations and identify developmental hypotheses that remain to be tested.

## 2. Review Scope and Literature Selection

This structured narrative review is organised by developmental stage and biological mechanism. “Structured” refers to this organisation and the explicit literature-selection approach, not to exhaustive evidence mapping or a systematic evidence synthesis. The original publication emphasis was January 2016 to July 2026, with earlier studies retained for receptor discovery, isoform characterisation, foundational genetic experiments, placental anatomy or concepts not adequately represented by later studies. Human studies and non-human mammalian models are considered separately before their findings are integrated.

In July 2026, a targeted literature search within PubMed and Embase was completed. Search terms included combinations of “angiogenic factors”, “physiology”, “sFLT-1 and PlGF”, “pre-eclampsia”, “fetal growth restriction”, “placental insufficiency” and “intra-uterine growth restriction”. Relevant titles, DOIs and reference lists were reviewed. Supplementary searches during revision were used to update the review and assess reviewer-suggested publications and replacement references.

The synthesis prioritises primary studies addressing placental development, ligand–receptor function, cellular or anatomical localisation, gestational variation, experimental manipulation or clinically relevant placental phenotypes. Reviews are used to position the present synthesis, not as independent confirmation of primary findings. Selection is purposive and based on mechanistic relevance. Evidence is compared according to study design, species, gestational stage and compartment, without meta-analysis or a formal risk-of-bias assessment. The review does not claim exhaustive coverage and remains susceptible to selection, publication and accessibility biases.

## 3. Molecular Framework of the FLT1–PlGF–VEGF System

VEGF-A signals predominantly through kinase insert domain receptor (KDR/VEGFR2) to promote endothelial proliferation, migration, survival and permeability. FLT1/VEGFR1 binds VEGF-A with higher affinity but generally weaker kinase activity, allowing it to function both as a signalling receptor and as a regulator of ligand availability [19]. The importance of this buffering role is demonstrated by disorganised vascular development in *Flt1*-null mice, whereas a receptor lacking the intracellular kinase domain permits substantially normal embryonic vascular patterning [20,21].

Alternative RNA processing of *FLT1* generates soluble proteins that retain extracellular ligand-binding domains but lack transmembrane and kinase regions. These include intron-13-derived sFLT-1 and the trophoblast-enriched sFLT-1-e15a isoform [22,23]. sFLT-1-e15a is abundant in the placenta, increases across gestation and inhibits VEGF-A-dependent KDR signalling [24]. A reported total sFLT-1 concentration therefore represents a mixture of molecular isoforms unless the assay is explicitly isoform-specific.

**Figure 1:**
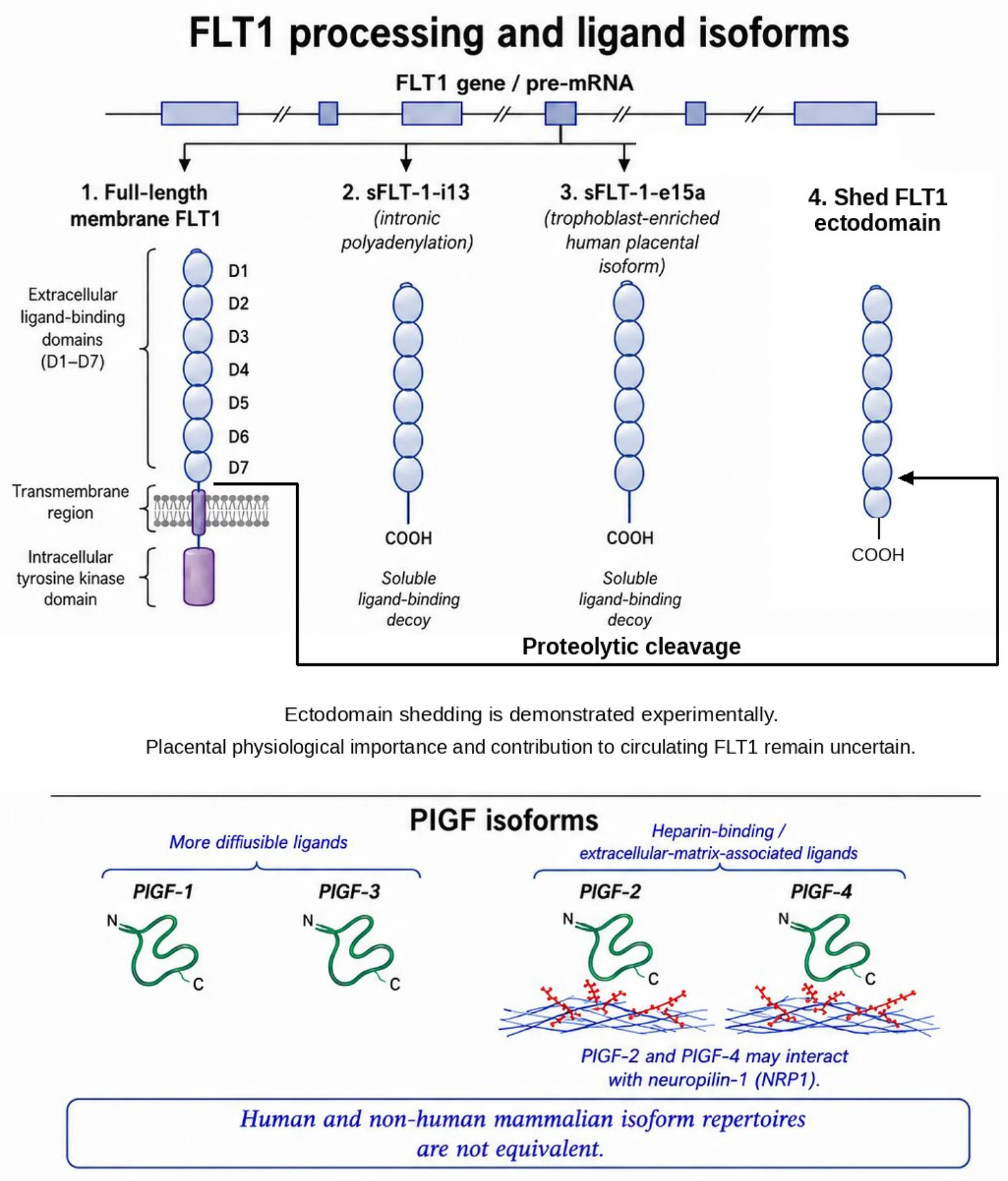
Alternative RNA processing, ectodomain shedding and PlGF isoform diversity. Alternative FLT1 RNA processing generates full-length membrane FLT1 and transcript-derived soluble isoforms (sFLT-1-i13 and sFLT-1-e15a). By contrast, the shed FLT1 ectodomain is shown arising from proteolytic cleavage of pre-existing membrane FLT1; the arrow from membrane FLT1 to the shed ectodomain denotes this cleavage step rather than alternative RNA processing. Shedding has been demonstrated experimentally [25,26], but its quantitative contribution to placental physiology and circulating FLT1 remains uncertain. PlGF isoforms are grouped according to diffusibility and extracellular-matrix binding. Abbreviations: FLT1, fms-like tyrosine kinase 1; sFLT-1, soluble fms-like tyrosine kinase 1; PlGF, placental growth factor; NRP1, neuropilin-1.

Separately, proteolytic cleavage of pre-existing membrane FLT1 releases a ligand-binding ectodomain. This post-translational shedding has been demonstrated in cell-based experiments and is distinct from the RNA processing that generates soluble FLT1 isoforms [25,26]. Its physiological importance in the placenta and its quantitative contribution to circulating FLT1 remain uncertain. These distinct routes and the PlGF isoform differences are summarised in Figure 1.

**Figure 2:**
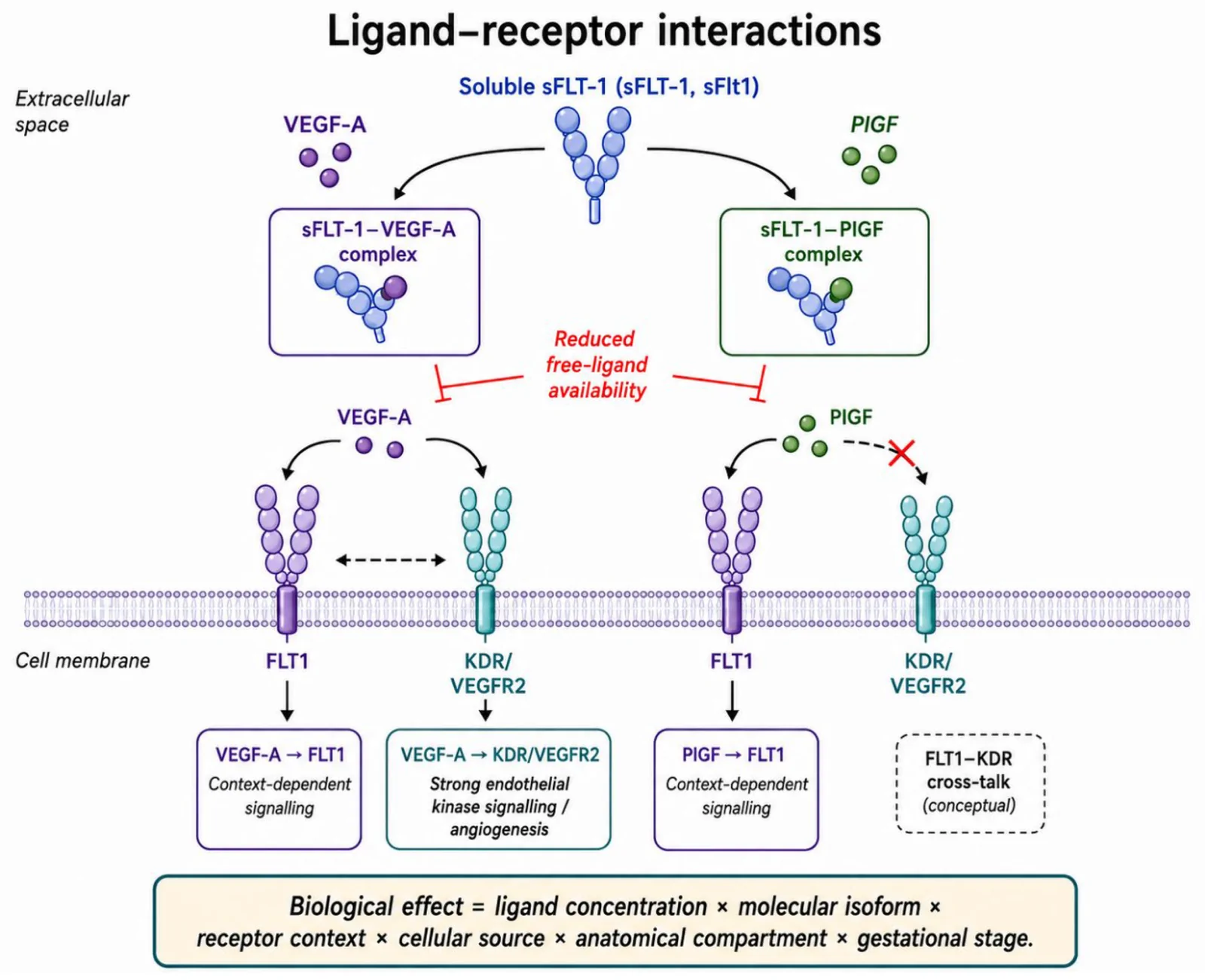
Context-dependent ligand–receptor interactions within the FLT1–PlGF–VEGF signalling network. sFLT-1 sequesters VEGF-A and PlGF, reducing free-ligand availability, while membrane FLT1 and KDR/VEGFR2 mediate distinct and interacting signalling pathways. Biological effects therefore depend on ligand concentration, receptor context, molecular isoform and anatomical compartment. The relationships are schematic; the multiplicative expression in the figure is illustrative, not a fitted quantitative equation. Abbreviations: VEGF-A, vascular endothelial growth factor A; FLT1/VEGFR1, fms-like tyrosine kinase 1/vascular endothelial growth factor receptor 1; KDR/VEGFR2, kinase insert domain receptor/vascular endothelial growth factor receptor 2; PlGF, placental growth factor; sFLT-1, soluble FLT1.

PlGF binds FLT1 but not KDR directly [4,5,27]. Its effects are consequently context-dependent: it can signal through membrane FLT1, redistribute VEGF-A away from FLT1 towards KDR, participate in receptor cross-talk or be neutralised by sFLT-1 [27]. Human PlGF isoforms also differ in extracellular-matrix binding, altering whether the ligand remains locally retained or enters the circulation [28]. The system is therefore better described as a compartmentalised ligand–receptor network than as a simple opposition between proangiogenic PlGF and antiangiogenic sFLT-1. The principal ligand–receptor relationships are summarised in Figure 2.

**Figure 3:**
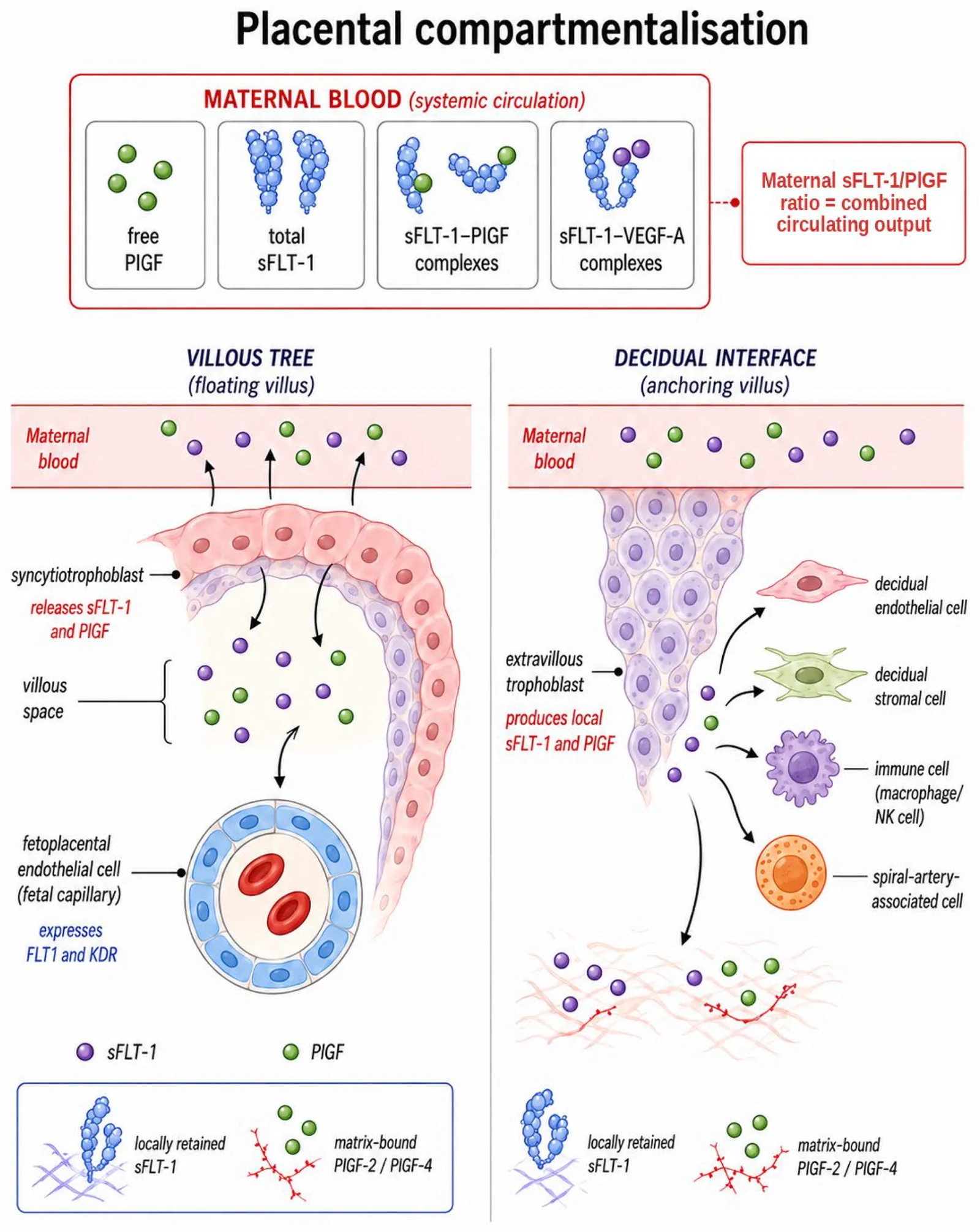
Placental compartmentalisation of sFLT-1 and PlGF signalling. sFLT-1 and PlGF arise from multiple placental compa rtments with different biological reach. Syncytiotrophoblast-derived factors can enter maternal intervillous blood and contribute to the circulating pool, whereas factors produced by extravillous trophoblast, decidual cells and foetoplacental endothelium may act predominantly through local autocrine or paracrine signalling. Matrix binding can further retain selected PlGF isoforms locally. The maternal sFLT-1/PlGF ratio therefore integrates the net effects of production, binding, sequestration, release and clearance across these compartments rather than directly measuring local receptor activity. Arrows indicate proposed routes of communication rather than measured fluxes; their relative contributions to maternal concentrations remain to be established. Abbreviations: sFLT-1, soluble fms-like tyrosine kinase 1; PlGF, placental growth factor; FLT1, fms-like tyrosine kinase 1; KDR, kinase insert domain receptor.

The anatomical location of the producing cell is a major determinant of the biological reach of sFLT-1 and PlGF. Because the syncytiotrophoblast is in direct contact with maternal intervillous blood, factors released from this compartment can readily enter the maternal circulation and exert endocrine effects. In contrast, sFLT-1 and PlGF produced by extravillous trophoblast, decidual cells and foetoplacental endothelial cells are more likely to act within local decidual or villous microenvironments through autocrine and paracrine signalling without a proportionate maternal serum signal [14,29]. Single-cell and spatial transcriptomic approaches have substantially improved the localisation of these interacting cell populations; however, conventional short-read sequencing generally cannot distinguish membrane-bound *FLT1* from individual soluble *FLT1* transcripts [22,23,30]. Their compartmental organisation is illustrated in Figure 3.

The maternal sFLT-1/PlGF ratio therefore reflects the combined circulating output of this network. It does not identify the producing cell, distinguish sFLT-1-i13 from sFLT-1-e15a, quantify membrane FLT1 or KDR activation, determine tissue VEGF-A availability or resolve diffusible from matrix-associated PlGF. This limitation does not reduce its clinical value; it defines the boundary between biomarker interpretation and tissue mechanism.

## 4. Comparative Framework for Mammalian Placentation

Mammalian placentas differ in gross shape, interhaemal barrier, exchange geometry and depth of trophoblast invasion [31]. The human placenta is discoid, villous and haemomonochorial; maternal blood directly bathes syncytiotrophoblasts, while extravillous trophoblast invades the decidua and uterine arteries. Non-human primates provide the closest experimental approximation to this architecture. Mouse and rat placentas are also haemochorial but labyrinthine and haemotrichorial, and they differ in trophoblast invasion and FLT1/PlGF isoform biology. Ruminant and porcine placentas retain maternal tissue layers and are most informative for blood flow, vascular expansion and exchange rather than human-like trophoblast invasion [31,32].

Direct trimester equivalence is not present between humans and any non-human mammal. Comparative transcriptomic analyses suggest that much of mouse placental development corresponds more closely to the first half of human pregnancy than to term [33]. Cross-species single-cell comparisons identify conserved invasive trophoblast programmes but greater divergence among syncytial lineages [32]. Models are therefore aligned with developmental events, including implantation, vascular transformation, exchange-region expansion or terminal maturation, and are chosen for the specific causal question rather than for overall anatomical resemblance.

## 5. Early Gestation: Implantation and Establishment of the Placenta

Early gestation encompasses implantation, decidualisation, trophoblast lineage formation, uterine vascular transformation and establishment of the maternal and foetal placental circulations. These processes overlap. Maternal blood flow into the human intervillous space develops progressively, while trophoblast plugs restrict flow and create a low-velocity environment during arterial remodelling [34,35]. Physiological low oxygen tension during this interval should therefore be distinguished from pathological ischaemia.

PlGF is present before a mature placenta forms. Human endometrial epithelium, decidualising stroma and leukocytes produce PlGF during the implantation window, and recombinant PlGF can modify blastocyst development and outgrowth in vitro [12]. After implantation, villous cytotrophoblast gives rise to syncytiotrophoblast and invasive extravillous trophoblast. First-trimester single-cell studies identify potential *PGF*–*FLT1* communication among trophoblast, endothelial, stromal and immune populations [36]. These predictions establish spatial plausibility but do not distinguish membrane FLT1 signalling from sFLT-1-mediated sequestration.

sFLT-1 is also part of normal early trophoblast biology. It is expressed in uncomplicated first-trimester syncytiotrophoblast and extravillous trophoblast and can be increased by macrophage migration inhibitory factor [13]. Experimental hypoxia increases trophoblast *FLT1* expression, including soluble isoforms, through hypoxia-responsive pathways [37]. This does not imply that normal first-trimester placentation is pathologically hypoxic; rather, oxygen-sensitive regulation provides a route through which prolonged low oxygen or malperfusion could exaggerate the antiangiogenic response.

Findings from mammalian models are consistent with an optimal-range interpretation, although no shared physiological set-point has been established. PlGF-deficient mice remain fertile but show altered uterine natural killer-cell differentiation, illustrating a local developmental role that is not captured by pregnancy viability alone [38]. Conversely, inadequate local sFlt-1 can permit excessive VEGF-A activity, while placenta-directed sFLT-1 excess suppresses uterine artery remodelling in a primate model [39,40]. Early placentation therefore requires sufficient VEGF-family activity for trophoblast–vascular communication, but also spatial restraint. The maternal circulating ratio does not reflect the spatial variation in these biomarkers.

## 6. Mid-Gestation: Placental Expansion, Vascular Branching and Exchange Capacity

Mid-gestation converts an established maternal–foetal interface into an organ capable of meeting rapidly increasing foetal demand. Villous tissue and the fetoplacental vasculature expand non-linearly, while the exchange barrier becomes thinner and capillaries are repositioned closer to syncytiotrophoblasts [41,42]. Exchange capacity depends not only on placental size or vessel density, but on maternal intervillous flow, foetal vascular resistance, barrier geometry, transporter activity and placental metabolism. Three-dimensional modelling confirms that oxygen and solute transfer are jointly constrained by vascular geometry, flow and diffusion [43,44].

The normal maternal angiogenic profile accompanies this expansion, but circulating concentrations do not establish local angiogenic activity. PlGF rises through the second trimester and peaks near 30–32 weeks, whereas sFLT-1 remains relatively restrained until later gestation [9]. Uterine venous studies confirm placental release of both factors, especially at term [10,11,45]. However, maternal concentration cannot determine whether rising PlGF reflects increased expression per cell, expansion of PlGF-producing tissue, altered release or reduced sequestration.

Low second-trimester PlGF is associated with lower placental weight, maternal vascular malperfusion, impaired foetal growth and preterm birth [46,47]. These associations identify developmental divergence but do not prove that reduced PlGF is the initiating lesion. Free PlGF may also fall because it is bound by sFLT-1 despite preserved total PlGF production [48]. In mice, *Pgf* loss changes placental compartment allocation and glycogen metabolism without abolishing foetal viability, illustrating functional redundancy and context dependence [49].

sFLT-1 may provide physiological vascular restraint during expansion; experimental overexpression demonstrates that excess can be harmful. Placenta-specific or trophoblast-restricted overexpression in mice alters placental differentiation, vascularisation, glycogen storage, nutrient-transporter expression and foetal growth [50,51,52]. Human foetoplacental endothelial experiments similarly indicate that total FLT1 supports migration and network formation while restraining proliferation [53]. Organised expansion therefore requires calibrated ligand availability rather than maximal angiogenesis.

## 7. Late Gestation: Maturation, Vascular Restraint and Placental Ageing

Late gestation is a period of active maturation rather than passive decline. Foetoplacental vessels continue to elongate and remodel, peripheral villi become more highly capillarised and effective diffusion distance is reduced [41,42]. At the same time, foetal demand increases faster than placental mass, making pregnancy increasingly dependent on the efficiency and reserve of the established exchange architecture.

The normal circulating profile shifts during this period: PlGF declines after its early-third-trimester peak, whereas sFLT-1 rises [9]. Increasing sFLT-1 may help restrain excessive VEGF-A activity, permeability and branching once the vascular network is established, but this physiological role remains inferential. The decline in free PlGF may reflect reduced production, altered trophoblast differentiation or increasing binding by sFLT-1 [48]. A rising ratio at term is therefore not intrinsically pathological; an early, marked or rapidly accelerating shift is more concerning for reduced placental reserve.

Senescence-associated features also accompany trophoblast differentiation. Human placentas show trophoblast-type-specific expression of senescence markers, including p16 and p21 [54]. ERVWE1-mediated cell-fusion experiments and observations of human syncytiotrophoblast link syncytial differentiation with senescence-associated pathways [55]. These findings should be distinguished from stress-induced placental ageing. Oxidative damage and senescence-associated features increase in post-mature and pathological placentas, while endoplasmic-reticulum stress can suppress *PGF* transcription and mitochondrial or growth-factor signalling can regulate sFLT-1 secretion [56,57,58,59].

A well-developed placenta can accommodate late foetal demand, whereas a placenta with earlier structural or perfusion limitations may decompensate as demand rises. Stress-related sFLT-1 release and reduced free PlGF may then amplify maternal endothelial dysfunction and further compromise placental vascular maintenance. The same final ratio can therefore reflect premature developmental failure, later demand–capacity mismatch or an acute stress response.

## 8. Evidence from Non-Human Mammalian Models

Experimental models are indispensable because human tissue is usually obtained after disease has developed and cannot be manipulated during an ongoing pregnancy. Their value, however, depends on matching the model to the causal question. Genetic deletion tests whether a pathway is required; placental overexpression tests whether local excess disrupts development; systemic administration tests maternal vascular effects; uteroplacental-ischaemia models test the response to impaired perfusion; and rescue studies test whether a downstream phenotype is reversible. These approaches should not be treated as equivalent reproductions of human pre-eclampsia.

### 8.1. Genetic Loss-of-Function Models

The foundational *Flt1* studies show why interpretation cannot be reduced to signalling strength. Complete *Flt1* deletion causes embryonic lethality with endothelial overgrowth and disorganised vascular assembly, whereas loss of the intracellular kinase domain permits much of normal vascular development [20,21]. The extracellular ligand-binding function is therefore essential for vascular patterning, while kinase-dependent signalling has more selective roles. Although these are embryonic rather than placenta-specific models, they establish the biological necessity of VEGF restraint.

PlGF-deficient mice provide valuable insights. Loss of *Pgf* alters uterine natural killer-cell differentiation [38], while separate experiments show changes in placental compartment allocation and glycogen metabolism without abolishing foetal viability [49]. In a catechol-*O*-methyltransferase-deficient model, PlGF loss also reduced maternal hypertension [49]. Thus, PlGF is a developmental modifier rather than an indispensable angiogenic switch, and its effect can differ between normal development and a specific pathological background. These findings argue against assuming that increasing PlGF will be uniformly beneficial.

### 8.2. Placenta-Directed sFLT-1 Gain of Function

Placental sFLT-1 overexpression models provide direct evidence that excessive local ligand sequestration can impair placental development. Placenta-specific sFlt-1 expression in mice reduces foetal and placental growth and alters spongiotrophoblast differentiation, glycogen-cell abundance and nutrient-transporter expression [50]. Inducible human sFLT-1 expression produces abnormal maternal and foetal vascularisation, disrupted labyrinth morphology and foetal growth restriction [51]. More restricted expression within spongiotrophoblast is sufficient to alter placental morphology, vascularisation and nutrient handling, including sex-dependent effects, even when maternal circulating human sFLT-1 is not markedly elevated [52].

These models distinguish local placental dysfunction from the maternal endothelial syndrome. A trophoblast compartment can be exposed to functionally important sFLT-1 concentrations while maternal blood shows only a modest change. Conversely, systemic sFLT-1 can produce hypertension and renal endothelial injury without reproducing the initiating placental lesion. The source and timing of sFLT-1 expression are therefore as important as the measured maternal concentration [51,52].

The primate model provides helpful insights into human uterine vascular transformation. Placenta-targeted sFLT-1 gene delivery to the baboon basal plate during early pregnancy produced a modest local increase in protein but substantially suppressed spiral artery remodelling [40]. This provides strong mechanistic evidence that excessive early sFLT-1 can disrupt vascular transformation within this experimentally manipulated primate model. It does not establish that endogenous sFLT-1 initiates all defective placentation, because the intervention imposed sustained expression and did not replicate the natural temporal and isoform distribution.

### 8.3. Uteroplacental-Ischaemia and Rescue Models

Reduced uterine perfusion in pregnant rats causes hypertension and increases circulating sFlt-1, supporting a sequence in which impaired perfusion can drive antiangiogenic signalling [60]. The model is useful for studying the downstream response to placental ischaemia, but the vascular restriction is imposed experimentally and cannot identify whether the initiating human lesion was inadequate trophoblast invasion, maternal cardiovascular limitation, thrombosis or another process.

Rescue studies strengthen causal inference. Recombinant PlGF abolished hypertension in the rat reduced-perfusion model, and PlGF reduced blood pressure in a non-human-primate model of uteroplacental ischaemia [61,62]. RNA interference directed against major placental sFLT-1 transcripts similarly reduced circulating sFLT-1 and improved disease features in mouse and baboon models [63]. These experiments show that ligand availability and placental sFLT-1 production are therapeutically tractable. They do not show that the initiating placental structure is restored, nor that the same intervention is appropriate at every gestational stage.

### 8.4. Mechanistic Conclusions from Experimental Models

Across species, the most consistent conclusion is that the FLT1–PlGF–VEGF system has an optimal rather than maximal setting. Insufficient FLT1-mediated restraint permits disorganised VEGF activity, whereas premature or excessive sFLT-1 impairs trophoblast differentiation, vascular transformation, exchange-region development and foetal growth. PlGF supports selected vascular, metabolic and immune functions, but its effects are partly redundant and context-dependent. The strongest mechanistic claims therefore arise from convergence across complementary models, not from any single species reproducing the whole human syndrome.

Translation requires attention to placental architecture and molecular orthology. Murine placentas do not reproduce human villous geometry, depth of trophoblast invasion or the complete human sFLT-1 and PlGF isoform repertoire. Non-human primates offer closer anatomy but smaller samples and fewer genetic tools. Large-animal models provide integrated haemodynamic and exchange measurements but differ substantially at the maternal–foetal interface. Model selection should be stated as an explicit link between biological question, developmental timing and compartment targeted. These complementary models and their principal causal inferences are summarised in Table 1.

## 9. Clinical Relevance Across Placental Disorders

The clinical value of sFLT-1 and PlGF is best considered in relation to placental vascular stress rather than a single disease. In pre-eclampsia, strong mechanistic evidence supports excess sFLT-1 as a mediator of maternal endothelial dysfunction: experimental elevation produces hypertension, proteinuria and glomerular endothelial injury, while human longitudinal studies show angiogenic changes before clinical presentation [6,7]. These findings do not establish excess sFLT-1 as the initiating lesion in every human pregnancy; increased release may follow placental injury and then amplify dysfunction [60]. Early- and late-onset phenotypes also differ in placental pathology and maternal susceptibility [64].

In PROGNOSIS, the primary analysis concerned women with singleton pregnancies and suspected pre-eclampsia at 24 + 0 to 36 + 6 weeks of gestation. In the validation cohort (*n* = 550), an sFLT-1/PlGF ratio ≤ 38, measured using Elecsys assays, had a negative predictive value of 99.3% (95% confidence interval, 97.9–99.9) for the absence of pre-eclampsia within one week [8]. This result is specific to the studied population, assay and time horizon; it does not establish 38 as a universal threshold throughout pregnancy or for FGR and other placental disorders.

In placenta-mediated FGR, low PlGF and an elevated ratio are associated with placental malperfusion and foetal deterioration, particularly in early-onset disease [46,47,65]. Human placental and serum studies suggest that reduced PlGF contributes more prominently to some FGR phenotypes, whereas increased placental sFLT-1 is more characteristic of the profile preceding pre-eclampsia [66]. Our BJOG systematic review found higher maternal ratios in pathological FGR than in constitutionally small pregnancies, but variable definitions and predominantly observational evidence support adjunctive rather than stand-alone interpretation [18]. Thus, similar ratios can arise through different changes in soluble-receptor release and ligand availability.

An antiangiogenic profile may also identify a placental vascular subset of foetal death, but the observational evidence does not establish universal stillbirth screening [67,68]. Other contexts include altered profiles in gestational diabetes [69] and the differential diagnosis of pre-eclampsia and active lupus nephritis [70]. These findings illustrate clinical overlap without implying a shared initiating mechanism.

Placenta accreta spectrum (PAS) provides a contrasting example: lower trophoblast sFLT-1 expression in invasive placentation is compatible with reduced local angiogenic restraint, but the tissue association does not establish that sFLT-1 deficiency causes PAS [71]. Its clinical importance extends to neonatal morbidity. In a retrospective cohort restricted to placenta praevia, histologically confirmed PAS was associated with greater neonatal respiratory resuscitation and supplemental oxygen requirements after adjustment for gestational age [72]. These outcomes reinforce the perinatal significance of invasive placentation, but they do not demonstrate a mechanistic link between reduced sFLT-1 and neonatal respiratory morbidity.

Clinical interpretation should therefore consider gestational timing, the relative contributions of sFLT-1 and PlGF, and the maternal–foetal phenotype. Biomarkers complement, rather than replace, maternal assessment, serial biometry and Doppler surveillance; they should not independently determine delivery or exclude all placental pathology. Representative patterns and interpretive cautions are summarised in Table 2.

## 10. Measurement and Interpretation

Apparent disagreement among studies often reflects differences in biological compartment, gestational age, assay and phenotype. Maternal serum, maternal plasma, decidua, villous tissue and foetal endothelium are not interchangeable. Total FLT1 does not distinguish membrane and soluble forms; total sFLT-1 does not resolve isoforms; and free PlGF is influenced by sFLT-1 binding [22,24,48]. Comparisons should therefore report specimen type, gestational age, assay platform, placental sampling site, labour exposure and clinical phenotype.

Each alteration should be considered as a potential contributor, compensatory response or marker. Experimental elevation of sFLT-1 provides strong mechanistic evidence for maternal endothelial and placental developmental effects within the tested models [6,50,51]. Human associations and temporal precedence alone cannot establish that sFLT-1 excess initiated the underlying placental lesion [7]. Conversely, impaired perfusion can increase sFLT-1 in experimental pregnancy [60], allowing a stress response to become a downstream mediator. Low PlGF has strong clinical and histological associations, but its functional contribution is context-dependent [46,49]. These interpretations are complementary rather than mutually exclusive.

Association must also be distinguished from individual predictive performance. In a retrospective cohort of 1164 pregnancies, lower first-trimester pregnancy-associated plasma protein-A (PAPP-A) and free β-human chorionic gonadotropin (free β-hCG) were associated with preterm birth and preterm prelabour rupture of membranes (pPROM), but individual-marker areas under the receiver-operating-characteristic curve remained below 0.70 [73]. This illustrates association without adequate discrimination, not direct evidence for FLT1–PlGF biology or a head-to-head comparison with the sFLT-1/PlGF ratio.

## 11. Integrated Framework and Future Directions

### 11.1. The Compartmentalised Angiogenic Rheostat: Evidence and Inference

We use the compartmentalised angiogenic rheostat as a qualitative organising framework, not an experimentally established model. The distinction between placental and circulating angiogenic activity is already recognised [15], as are physiological, diagnostic and therapeutic applications [16,17]. Unlike our diagnostic systematic review [18], the present synthesis links these established components across developmental stages, isoforms and species. “Rheostat” is a metaphor for context-dependent regulation; it does not imply that a placental set-point or quantitative response function has been measured.

The supporting evidence falls into different categories. Human longitudinal and uteroplacental sampling studies establish gestational trajectories and placental release [9,10,11,45]; tissue, spatial and isoform studies establish molecular diversity and anatomical expression patterns [14,22,23,24,27,28,29,30]. They do not directly measure all local ligand fluxes. Genetic deletion, targeted overexpression and rescue studies establish effects of perturbing the pathway in specific experimental systems [20,21,38,39,40,50,51,52,60,61,62,63]. The proposed integration of these observations into a gestation-dependent regulatory framework, and its application to individual human disease trajectories, remain hypotheses.

The first dimension is time. Human studies demonstrate changing maternal concentrations and placental architecture across gestation [9,41,42]. Experimental models show that disturbing ligand availability can alter implantation-site immune cells [38], disrupt vascular transformation [39,40] and impair later placental growth [50,51,52]. These findings support attention to developmental timing, but do not establish a single optimal concentration for each stage. In particular, a protective late-gestational role for rising sFLT-1 in limiting permeability or branching remains inferential.

The second dimension is space. Human tissue studies identify distinct decidual, trophoblast and endothelial environments, while uteroplacental sampling confirms release into maternal blood [10,11,14,29,45]. Local perturbation studies show that placental effects need not be proportional to maternal concentrations [40,52]. Matrix binding provides a mechanism for local PlGF retention [28], but the contribution of each compartment to an individual maternal ratio remains unresolved. Consequently, local activity and systemic biomarker behaviour cannot be treated as interchangeable.

The third dimension is molecular form. Human molecular and functional studies distinguish membrane FLT1, soluble transcripts and PlGF isoforms, and demonstrate differences in ligand binding and receptor interactions [22,23,24,27,28]. These are established components of the framework, not new mechanistic claims. However, their combined, gestation-dependent effects in intact human placental compartments are incompletely characterised; total gene or protein measurements cannot resolve them.

The fourth dimension is placental architecture and maternal–foetal context. Species differ in exchange geometry, invasive lineages and isoform repertoire [31,32,33]. An intervention in a murine labyrinth or primate placental bed therefore tests a specific developmental question, not the entire human syndrome. Maternal susceptibility and foetal demand may further modify the clinical consequences of a placental signal [64], but are not quantified by the circulating ratio.

The framework helps interpret, rather than experimentally resolve, several apparent paradoxes. FLT1-mediated ligand restraint is necessary for organised embryonic vascular development in genetic models [20,21], whereas excessive soluble FLT1 can impair placental development [50,51,52]. This distinction avoids attributing the entire *Flt1*-knockout phenotype specifically to soluble isoforms. PlGF effects can also differ by experimental context [49]. A high maternal ratio may therefore be clinically informative without specifying the underlying local mechanism.

Four non-exclusive developmental trajectories can be hypothesised: early failure of vascular transformation with subsequent malperfusion; mid-gestational limitation of placental growth and ligand availability; late mismatch between foetal demand and placental reserve; and acute placental stress without a prolonged preceding biomarker change. These trajectories integrate the preceding evidence but are not validated disease subtypes, nor can they be assigned from the ratio alone. Longitudinal sampling linked to spatial tissue measurements is required to distinguish them.

The framework is intended to generate testable questions: which cell and isoform change, whether the effect remains local or becomes systemic, and whether the alteration precedes injury, compensates for it or amplifies it. Its main contribution is this integration and explicit separation of evidence from inference, rather than the proposal of a newly proven regulatory system.

### 11.2. Priorities for Research and Translation

The highest priority is to link longitudinal maternal trajectories to spatial placental biology. Serial circulating measurements should distinguish free from total PlGF; complementary tissue studies should quantify individual sFLT-1 transcripts wherever possible, while incorporating placental imaging, foetal growth, Doppler haemodynamics and standardised histology.

Human trophoblast organoids provide an experimental foundation; adding endothelial, stromal and immune compartments, controlled oxygen and perfusion could help test specific hypotheses within this framework [74,75]. Animal studies should use inducible, cell-specific manipulations aligned to the developmental event under investigation. Clinical pilot studies of circulating sFLT-1 removal [76] complement experimental PlGF supplementation and placental sFLT-1 silencing [61,63]. Recombinant VEGF121 infusion also improved maternal blood pressure and renal function in a rat model of reduced uterine perfusion [77]. These studies demonstrate pathway-directed proof of concept rather than restoration of a complete regulatory framework. Translation must preserve the physiological FLT1 and VEGF functions required by maternal, placental and foetal tissues.

## 12. Conclusions

sFLT-1 and PlGF are gestation-dependent regulators and indicators of placental biology, not simply opposing biomarkers of pre-eclampsia. Human observations establish gestational variation, molecular diversity and placental release; experimental perturbations provide strong mechanistic evidence for effects on vascular and placental development. The compartmentalised angiogenic rheostat integrates these findings as a working framework, while local physiological set-points and proposed human disease trajectories remain unvalidated. The maternal sFLT-1/PlGF ratio is most informative when interpreted within the clinical and gestational context, not as a direct measurement of tissue signalling or proof of an initiating lesion. Future progress requires longitudinal, isoform-resolved and spatially informed studies that connect the circulating biomarker to the placental processes it reflects.

## Figures and Tables

**Table 1:** Selected mammalian models and the causal questions they address.

Model	Principal Finding	Causal Inference	Key Limitation
*Pgf*-deficient mouse [38,49]	Altered uterine natural killer-cell differentiation, placental compartment allocation and metabolism without complete pregnancy failure	PlGF modifies development but is partly redundant and context dependent	Murine PlGF isoforms and labyrinthine placentation differ from humans
Placental/trophoblast sFLT-1 overexpression in mouse [50,51,52]	Abnormal differentiation, vascularisation, nutrient transport and foetal growth	Excess local sFLT-1 can directly impair placental development	Expression level, cell target and human transgene may be non-physiological
Placenta-targeted sFLT-1 in baboon [40]	Suppressed uterine spiral artery remodelling	Early local sFLT-1 excess can disrupt primate vascular transformation	Small experimental groups and imposed sustained gene expression
Reduced uterine perfusion with PlGF or RNAi rescue [60,61,62,63]	Ischaemia raises sFlt-1; restoring ligand availability or reducing sFLT-1 improves maternal features	The antiangiogenic pathway is a modifiable mediator downstream of placental stress	The initiating lesion is experimentally imposed; restoration of placental structure is not established

**Table 2:** Clinical interpretation of selected angiogenic patterns [6,7,8,9,18,46,47,48,49,65,66,67,68,69,70]. Biological interpretations are hypotheses informed by the cited evidence, not mechanisms established by the ratio alone.

Clinical Pattern	Possible Biological Interpretation	Most Relevant Contexts	Caution
Markedly high sFLT-1 with low free PlGF	Excess soluble receptor release and ligand sequestration	Pre-eclampsia; severe early placental FGR	Does not identify the initiating placental lesion or maternal susceptibility
Predominantly low PlGF	Reduced production, placental mass or trophoblast differentiation; may also reflect binding	Some FGR phenotypes and placental malperfusion	Free PlGF is not equivalent to total placental production
High ratio without hypertension	Placental vascular dysfunction with predominantly foetal consequences or preclinical maternal disease	Early FGR; selected foetal-death phenotypes	Requires foetal surveillance even if maternal criteria for pre-eclampsia are absent
Normal or modest ratio with concerning clinical findings	Non-angiogenic pathology, late/atypical disease or constitutional smallness remains possible	Chronic hypertension, renal disease, foetal vascular or genetic causes	A normal result should not override clinical, laboratory or Doppler evidence

## Data Availability

No new data were generated or analysed in this study. All information discussed is derived from the published literature cited in the manuscript.
